# Evolution in Dentistry

**Published:** 1890-02

**Authors:** J. A. Robinson

**Affiliations:** Jackson, Michigan


### Evolution in Dentistry.

PERSONAL EXPERIENCE, BY J. A. ROBINSON, D.D.S.,
JACKSON, MICHIGAN.

As I have worked at the teeth probably more days and years than any person now living, I propose to gratify some of my personal friends (at their request) and also to show as well as I can some of the difficulties that have been surmounted by myself and others in climbing a rough and rugged way to our present degree of standing and excellence as a profession. I trust you will forgive so frequent mention of my own life, as it seems necessary to illustrate my connection with my life-work; as I have been doing something in dentistry, in a small way, almost from my boyhood.

I was born and raised in Old Concord, Mass., born May 31, 1812. A lameness in my left ankle made it necessary to go about upon crutches more than a year about the time I was twelve years old. Being obliged to sit around the house a good deal, I employed my time in whittling dolls out of pine wood for my two sisters, one younger and one older than myself. As the year I was away from school, from my lameness, put me behind the other boys in my studies, I disliked my school, and on account of feebleness, I was apprenticed to Joshua Haynes, who was a watch maker in the village, the day I was fourteen years old, and was to remain with him seven years—seven years was the term at that time for all mechanical work. My apprentice life was not a pleasant one to me. I did not like my master, as Mr. Haynes was called, because I was a bound apprentice boy. The work of making the various parts of a watch was very much to my taste (for we made watches), but Mr. Haynes followed his gun and dogs in the hunting season a good share of the time, and left the shop to one Joel Sawyer, an older apprentice than myself, to look after the work. In those days every person drank more or less New England rum, and Mr. Haynes among the rest. He was not a drunkard, but as Burns would say "I was not fou, but just had plenty." So Mr. Haynes had just plenty to make him difficult to get along with, and sometimes very cross. When he was in the shop he was all right. He knew a great deal of all kinds of mechanics, was a chemist in a small way, and was a natural teacher; so we got along well when he was at work. He delighted in all manner of chemical experiments, and we had an old "Black's Chemistry" that was authority in all metallurgy at that time, also compounding metallic substances and various alloys that occupied all our spare time, and I was taught to refine gold and silver, and do gold and silver plating, moulding and casting various metals, and harden and temper steel, and forge any little steel or iron thing that was necessary to be had about a shop, I did almost every thing. Dentistry at that time was simply mechanism and adaptation.

The greatest contention I had with my master was about my fiddle; I was fond of playing a violin, and playing for the boys and girls to dance at the various houses in the village two or three nights in a week, while Mr. and Mrs. Haynes wanted me to go to prayer meeting instead of dancing. The friction between us grew worse and worse until I ran away and left home. Then came the trouble with my father and mother. Mr. Haynes insisting that I was valuable to him as an apprentice, that I owed him labor, was faithful as a worker and a good boy all except my fiddling. The fiddle I never could abondon and never did, but went back to work. When about sixteen years old I became acquainted with an old man by the name of Baker, who came around about twice a year to make teeth for the old ladies who needed his services.

The first set of artificial teeth I ever saw were worn by Dr. Baker himself. They were made of calf's teeth and the teeth of sheep fastened on a piece of thick leather and worn under the upper and under lips, and were only worn for show and taken out of the mouth while eating. He also made some sections of six or eight front teeth out of bone that were tied in with ligatures to the back teeth. A thighbone of an ox was boiled and scraped and a section sawed off about the length of the natural teeth and of the size to fill the place of the missing teeth and tied in with a silk cord, after being filed down into sections to resemble the teeth. About a year after Dr. Baker's visit to Concord came a Dr. Dewar, of Boston, a French dentist, and he brought with him human teeth said to have been taken from the battlefield of Waterloo, and preserved in alcohol, and set them on the roots with a wooden pin or dowel. This was about the A. D. 1828. There were no dental instruments to be bought, and of course, they were all home-made, by the blacksmith, or some general tinker who makes every thing in a small town. As I had poor teeth for a boy, and was something of an expert in the use of tools, having learned to make the wheels and pinions of watches and to temper steel, I was employed to manufacture Dr. Dewar's dental instruments, or tools as they were called at that time. I got shoemakers' awls to make the small excavators, pluggers and burrs, and put them into ivory or wooden handles, and made a sort of outfit for Dr. Dewar in that way, and he filled my teeth for doing the work. I spent a good deal of time with Dr. Dewar and saw him set a good many human teeth on wooden pivots. After Dr. Dewar left Concord and went back to Boston I began to make a few partial sets out of bone, and got some sea horse tusks, as it was finer ivory, and I made a number of small pieces for the toothless women in the town that did good service for a number of years. I also made quite a respectable turnkey that I have in my possession to-day. There was no instrument for extracting teeth but a turnkey, and Flagg's forceps were invented and first used about 1832 or '33 and were not put on sale till 1836. In the financial crisis of 1835 I began dentistry by reading medicine with Dr. George Mansfield, of Lowell, Mass., who was a pupil of Harwood & Tucker, Hamilton Place, Boston. Mansfield was an M.D. and had not been in practice long at dentistry. The only dental work was Bell on the Teeth.

As my former business and little experience in dentistry had prepared me somewhat for a change in business, and the use of tools and working in gold and silver and other metals had given me some experience in tinkering and doing almost every thing, I soon became a strong help to Mansfield, as he made artificial sets of teeth on gold and silver plates, so at the end of the year he complimented me by saying to old Dr. Flagg that I had taught him more than he had taught me. My contract with Mansfield for instruction was to be two hundred and fifty dollars. One hundred and fifty I paid on entering his office, and to show his appreciation of the service I had been to him he gave me the one hundred dollars due as a donation.

While with Mansfield we began the manufacture of mineral teeth after the plan of the old Stockton tooth, which was a compound of pipe clay, ground to impalpable powder, and feldspar for the base or body, with enamel of feldspar and a little chalk and shaded or colored with titannum for yellow, and cobalt for coloring of blue enamel for the cutting edges of the teeth. These teeth were very strong, but very opaque, and resembled white beans about as nearly as human teeth. It will be unnecessary for me to go all through the series of experiments we made before we found a translucent material to make a tooth fit to be worn. The best material was selected from a pile of paving-stones. It is enough to say that it involved stones out of the street—a small boulder of conglomerate quartz—a good deal of hard labor in grinding, and feldspar—a sort of graphite granite that was vitrified—the material and coloring for the enamel that was ground fine and powdered in lead moulds.

My attainments in carving block work I gained by studying natural models. With an old skull in my hands I sat for hours and hours studying the form, shape and size of the human teeth until I understood the organization, articulation, form and size of each individual tooth of the superior and inferior maxillaries, called then the upper and lower jaws. This was good discipline, for every thing is dual, and we all have to make whatever we desire to make perfect within ourselves first, before we can reproduce it outside of ourselves. So the whittling propensity and practice of my boyhood was useful to me when I wanted to make teeth, or models for moulds to manufacture block work or single teeth. As I began dentistry with a horse and wagon and traveled from house to house, and from town to town, I was astonished at the amount of secrecy that was demanded of me from my patients, almost every one exacted a promise from me that I would not let it be known in the neighborhood that Mrs.

or Miss A or B had a false tooth or a set of teeth, and even when I settled in Old Salem, Massachusetts, many times, ladies have refused to give me their names for the appointment, saying "Now please don't mention to any person that I am having any new teeth, for I would not have any one know it for the world." And these same persons would come disguised or with a thick veil to cover the face to see the dentist for fear of being seen, or ask if they could not come in by the back door. In the summer of 1836 I made an upper set of teeth for the mother of Ralph Waldo Emerson, who was a relative of my mother, and I made the *plate* out of a section of ivory I got at a piano factory in Boston. I got an impression in beeswax and filled it with soft putty, dried it thoroughly and that was the only model I had to make the inside of the plate fit the mouth. I cut out the plate on the inside with chisels and small gouges and used a pigment of red lead to guide me as to the shape, and the irregularities and depressions; then I filed down the outside of the plate to about one-quarter of an inch in thickness and set on the ivory plate ten *human teeth* with wooden dowels and this plate was worn up to the time of her death and gave good satisfaction. Of course the fit was very imperfect, but as Dr. Flagg used to say if the patient could answer questions in monosyllables without the plate coming down into the mouth it was all he could expect.

While going about in the towns in search of business, if I found a patient in a tavern I made a dental operating chair by standing on one foot and placing the other foot in a chair back of the person and resting his head against my knee to steady it while I cleaned and filled his teeth, and if I visited the house to work for the women folks I sat down on a low cushion and had the persons sit on the floor and place their heads against my left arm for a head rest while I operated on their teeth.

Sometimes my lady patients would remark that this was "a very awkward and singular position to be placed in" to have their teeth fixed, but as we had no other conveniences it was made to answer the purpose of the dental chair.

In making partial sets of teeth out of bone or ivory we paid no attention to the articulation, but left a shoulder on the under side to touch lightly on the lower teeth, and if there were any number of back teeth it was the rule not to have the block of bone touch the lower teeth at all.

Dental operations at that time were purely mechanical. The only regulating was to extract superfluous teeth and give nature an opportunity to correct herself. It was the Harwood and Tucker plan to extract alternate bicuspids and molars that were badly decayed to make a good free opening to fill an adjoining tooth saying, that ten or twelve good sound and healthy teeth were more useful than a full set of artificial teeth, and it was more practical than to try to fill teeth that were of a doubtful character, and their establishment was the leading dental place in Boston or New England.

As dentistry was purely a mechanical attainment in the beginning, professional etiquette was unknown, and the dental laboratory was a sealed enclosure, a secret place with "*No admittance*" placed over the door.

There was a small number of men in Boston who visited together and compared notes, and showed samples in the study and improvement of the manufacture of "mineral teeth," but when Dr. Harwood succeeded in producing the best samples he differentiated, as the scientist would say, that is, he refused to divulge his secret and branched off to himself, and that broke up the institution.

I gained admission to Harwood and Tucker through Joshua Tucker, who married a Miss Morse, of Winchendon, and her brother, Milton Morse, married my cousin, so Tucker said I was his cousin and could come in.

It is a hazardous task to light up the coldness and treatment the country dentist received from those dentists who were well situated in Boston fifty years ago. As I stated before, dentistry was purely mechanical and the evolution in mechanics is the new method of the old plan that was true. This can be illustrated by the germ of the original pivot-tooth as compared to the Richmond crown, or the dental engine compared to the old method of finishing fillings, or taking impressions in plaster instead of beeswax so the fit would be perfect enough to dispense with the gold spiral springs for full sets of teeth to keep the upper and lower dentures in place. Difficulties always suggest remedies, a profession grows higher and broader as we grow and improve in intelligence and culture.

